# Efficacy of Pegylated Hyaluronic Acid Filler Enriched with Calcium Hydroxyapatite: A 24-Week Post-Market, Observational, Prospective, Open-Label, Single-Center Study

**DOI:** 10.3390/jfb14070345

**Published:** 2023-06-29

**Authors:** Nicola Zerbinati, Edoardo D’Este, Annalisa De Silvestri, Marco Zullino, Giulio Rabbiosi, Stefania Guida, Paweł Kubik, Giorgio Stabile, Roberto Mocchi, Chiara Barlusconi, Sabrina Sommatis, Giovanna Cipolla

**Affiliations:** 1 Dermatologic Unit, Department of Medicine and Surgery, University of Insubria, 21100 Varese, Italy; 2 Dermatologic Unit, Centro Medico Polispecialistico, 27100 Pavia, Italy; 3 Unit of Clinic Epidemiology and Biometric Scientific Direction, Foundation IRCCS Policlinic San Matteo, 27100 Pavia, Italy; 4 Department of Mathematics and Applications, University of Milan Bicocca, 20125 Milano, Italy; 5 Meidicine and Surgery Department, Università Vita-Salute San Raffaele, 20132 Milan, Italy; 6 Dermatology Clinic, IRCCS San Raffaele Scientific Institute, 20158 Milan, Italy; 7 Centrum Medyczne dr Kubik, Skwer Kościuszki 15/17, 81-370 Gdynia, Poland; 8 UB-CARE S.r.l.-Spin-Off, University of Pavia, 27100 Pavia, Italy; 9 Department of Biomedical Sciences and Human Oncology, Section of Dermatology, University of Bari, 70121 Bari, Italy

**Keywords:** pegylated hyaluronic acid filler, calcium hydroxyapatite, facial aging

## Abstract

Recently, thanks to the greater discovery of the mechanisms of facial aging, an alternative to invasive plastic surgery has found space with less invasive aesthetic procedures, also based on an increasingly pressing request. We are specifically referring to dermal filler injection into or under the skin which leads to immediate rejuvenation and aesthetic improvements. In this study, we wanted to analyze the results obtained through the use of NEAUVIA Organic Stimulate, particularly with regard to its effectiveness, which is a cross-linked polymeric hydrogel, containing stabilized sodium hyaluronate 26 mg/mL and calcium hydroxyapatite (1%), glycine and L-proline in buffer pyrogen-free water, in its main indication, namely, the temporary correction of congenital and acquired deficiencies of the soft tissues of the face by intradermal injection. Initially, 70 patients were enrolled, but 10 did not complete the study due to non-observance of the investigation rules, so they were excluded from the protocol. The collected data demonstrate an efficient mechanical effect of the pegylated polymeric acid matrix enriched with low concertation of calcium hydroxyapatite and in accordance with other evidence in vitro and in vivo, and the mechanical support of the interstitial connective space improves the homestays of the anatomical layer rebalancing the physiological activity of the dermis cells.

## 1. Introduction

Recently, thanks to the greater discovery of the mechanisms of facial aging, an alternative to invasive plastic surgery has found space with less invasive aesthetic procedures, also based on an increasingly pressing request. Today, minimally invasive non-surgical actions are the techniques in greatest request, for better acceptance and tolerance, to reduce facial wrinkles and improve facial volume and contours.

We are specifically referring to dermal filler injection into or under the skin which leads to immediate rejuvenation and aesthetic improvements; when used correctly, dermal fillers offer excellent clinical results with minimal-to-no downtime [1,2,3]. The changes inherent in the volume of soft tissues and bones contribute significantly to a change in facial appearance compatible with age. The decrease in bone tissue and soft tissue thickness, the consequent redistribution of fat, in addition to the reduction of elasticity and relief of the skin lead to the establishment of wrinkles and folds that characterize the signs of aging. This succession of events often occurs within the age of 30 in a fair number of people. In the mid-face, flattening, and furrowing of the central mid-cheek area are observed, with medial displacement resulting in an exaggerated depth of the nasolabial folds [4].

There are now more treatment options with the availability of soft tissue fillers and associated developments in injection techniques. Dermal fillers were first used to improve skin tone, firmness, and texture as part of surface line and wrinkle management. Subsequently, the indication for the treatment of volume deficits, by means of subcutaneous and deep injections, was also outlined, using a vast range of articles designed in a specifically targeted way [5,6]. Additionally, patients also experienced associated improvements in psychosocial function [7,8]. Collective adverse events associated with filler use include infection, allergic reaction, and swelling. Rarer situations include the triggering of autoimmune reactions, visual disturbances, blindness, and stroke [9]. Physicians using dermal fillers should be able to recognize potential complications and know their appropriate and timely management [10,11]. Different injectable products have very different properties, associated risks, and injection requirements. Hyaluronidases are endoglycosidases, already physiologically present in the human body, which break down HA by decreasing its viscosity. Hyaluronidase can degrade HA hydrogels and can prevent severe vascular complications. For this reason, the immediate availability of hyaluronidase is essential for any doctor using HA fillers [12,13]. Injectable fillers have a wide range of medical indications. Clinically, fillers can correct subcutaneous fat atrophy from human immunodeficiency virus (HIV) lipodystrophy or progressive hemifacial atrophy. Facial lipoatrophy (FLA) is a stigmatizing feature of HIV-infected patients and can put their quality of life at risk, also leading to severe psychological discomfort, compromising their body image, loss of self-esteem, depression, social isolation, sexual dysfunction, and occupational barriers [14,15]. In addition, dermal fillers find further utility in correcting facial defects resulting from traumatic facial injuries. Among people aged 18 to 45, there has been an estimated 20% incidence of facial trauma directly related to traffic accidents, sports injuries, assaults, or falls [16]. Invasive surgical therapy in this type of patient is often complicated and very demanding and is not free from risks related to unfavorable functional and aesthetic results that end up compromising the patient’s quality of life. Many treatment techniques have been refined in recent years to restore facial volume and improve the appearance of scars [16,17,18]. These include dermal fillers, autologous fat grafts, lasers, and dermabrasion. Hyaluronic acid (HA) fillers, among the most recent as dermal fillers, are currently considered the “gold standard” as they contain precisely the ideal characteristics mentioned. Indeed, HA is a biopolymer already present in nature, specifically concentrated in the extracellular matrix of the soft connective tissue, in the skin dermis, in the vitreous body of the eye, in the hyaline cartilage, in the synovial joint fluid, and the disc nucleus and the umbilical cord. In cross-linking, HA reacts with a cross-linking agent which can create covalent bonds between HA chains. Therefore, the chemical cross-linking of HA is essential to prolong its residence time in the dermis [19]. HA is commercially available in various pharmaceutical forms, such as nanoparticles, nanocomplexes, matrices, and hydrogels [20]. Chemical and biochemical characterization of hydrogels was, accordingly, performed with several different aims, ranging from safety assessment, and quality assurance to the understanding of hydrogel properties (rheology, degradation, and fitness for purpose) [21,22]. Specifically, the cross-linking parameters play a vital role in determining the rheological and swelling properties of the hydrogel characteristics which are of utmost importance for clinical applications [23].

In this study, we wanted to analyze the results obtained with NEAUVIA Organic Stimulate, which is a cross-linked monophasic polymeric hydrogel, containing stabilized sodium hyaluronate 26 mg/mL and calcium hydroxyapatite (1%), glycine and L-proline in buffer pyrogen-free water, with a modification degree ranges from of 6.2% and with an effective crosslinker ratio of 0.07. The main indication of the medical device is the temporary correction of congenital and acquired deficiencies of the soft tissues of the face by intradermal or subcutaneous injection. Neauvia hydrogels are based on PEGDE (polyethylene glycol diglycidyl ether) cross-linking technology which appears to have better biocompatibility, excellent biointegration, and optimal rheological characteristics [21,22,23]. PEG has a very high safety profile and toxicity that is considered lower than other cross-linking agents used up to now. In fact, it is reported to have a peculiar and extremely interesting characteristic, namely, that of concealing the agent to which it binds by the host’s immune system, with consequent reduction of immunogenicity and antigenicity [24,25,26]. The goal of this paper is precisely to estimate the effectiveness of NEAUVIA Organic Stimulate in the use of minimally invasive aesthetic medicine.

## 2. Materials and Methods

### 2.1. Study Design and Subjects

This is a post-market, non-profit, observational, prospective, real-life, open-label, single-center, study. The observational design enabled the evaluation of patients in a broad range of settings reflecting routine clinical practice. All decisions about procedures, treatments or use of resources were at the discretion of the treating physician. The aim of the complete study was to assess the efficacy and the safety of the cross-linked PEG hydrogel hyaluronic acid-based injectable implant (Neauvia Organic Stimulate, Matex Lab Sa—Geneve-Switzerland) for the treatment of facial soft tissue deficits, in particular, it concerns the correction of the central part of the face, in order to be able to determine the risk/benefit profile of the product itself.

**Figure 1:**
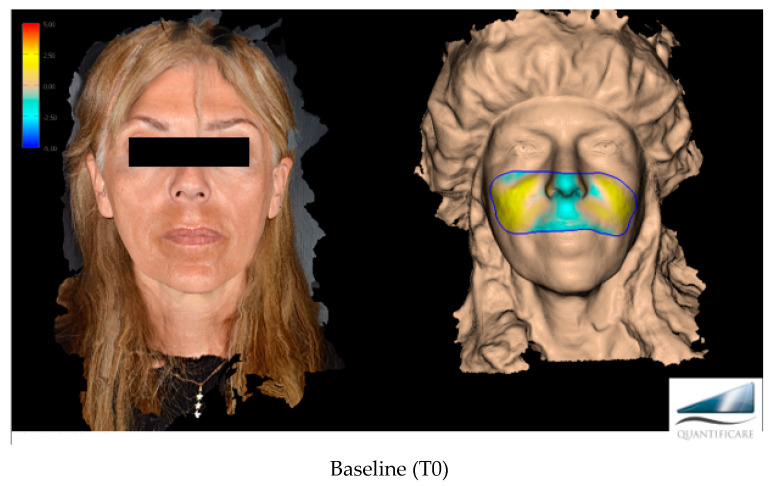

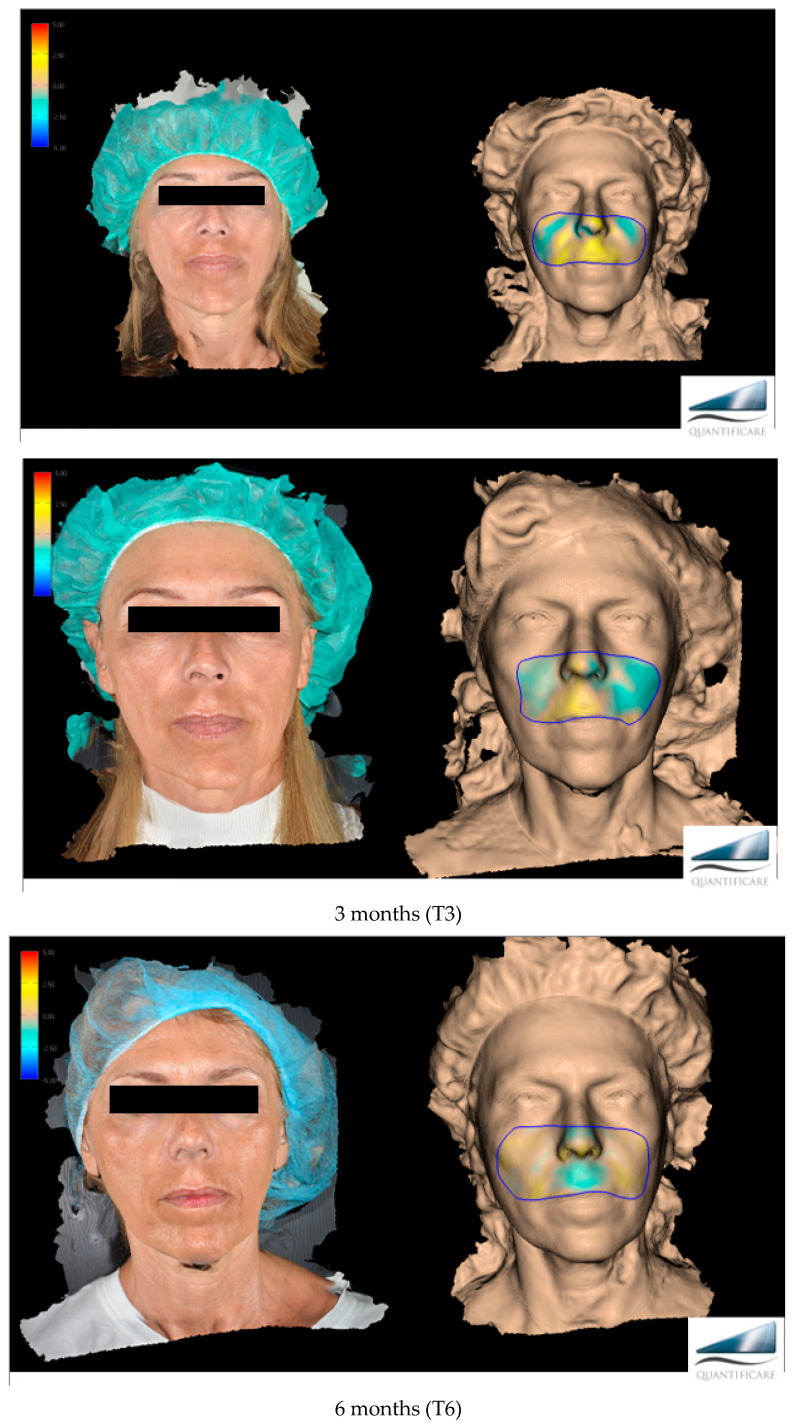
Before and after evaluation in the different time points with a 3D photographic system (LifeViz^®^ Mini by Quantificare).

The primary target of this work, more specifically, was to evaluate the effectiveness of Neauvia Organic Stimulate dermal fillers. Clinical efficacy was estimated based on images taken by the investigator, comparing pre-treatment images with post-treatment images (Figure 1), but for the real clinical considerations of efficacy, two evaluation scales were used: the mid-face volume deficit scale (MFVDS) [27] and the Global Aesthetic Improvement Scale (GAIS) [28].

Upon completion of the global assessment, investigator and subject satisfaction with the aesthetic result was determined using the VAS scale [29,30].

The study protocol was submitted to an independent Ethics Committee for review and written approval (Ethical Committee of Pavia, Italy) (Record number: P-20200010554 protocol number: 20200114610).

The study was conducted in accordance with the protocol of the International Conference on Harmonization (ICH) and Good Clinical Practice (GCP) and the Declaration of Helsinki. In addition, the study was conducted in compliance with all applicable local and international laws and regulatory requirements relevant to the use of medical devices. The study was carried out at the Centro Medico Polispecialistico of Pavia, Italy.

### 2.2. Selection of Study Population

A number of 70 patients were enrolled, but only 60 fell within the total parameters of the study protocol. Fifty-six were female (93.33%) and four were male (6.67).

Patient inclusion criteria were:

- To be men or women, at least 18 years of age but not more than 70 years of age at enrollment;
- To have a reasonable potential for benefit from correction;
- To have a moderate to severe age-related mid-face volume deficit according to the mid-face volume deficit scale (MFVDS) [27];
- To be able to understand and comply with the requirements of this study;
- To be willing and able to provide medical history and informed consent prior to any study-related procedures being performed;
- To be willing to comply with all aspects of the treatment and follow-up schedule and procedures.

Patient exclusion criteria were:

- To be pregnant, lactating, or trying to become pregnant;
- To be children or teenagers;
- To have had prior therapy (e.g., other permanent or biodegradable injectable fillers or surgical correction) within 3 months before the HA injection;
- To have had previous tissue augmentation with permanent implants (e.g., silicone) in the area to be treated;
- To have any active inflammation, infection (acne, herpes, dermatitis, etc.) or unhealed wound of the face;
- To have varices in the area of the implant;
- To have auto-immune disorders affecting the skin;
- To undergo radiation or ultrasound therapy in the area of the implant;
- To have a known hypersensitivity to the test product (hyaluronic acid or its ingredients);
- To tend to develop hypertrophic scarring;
- To suffer from untreated epilepsy;
- To have a history of anaphylaxis or history of severe allergies;
- To use simultaneously laser treatment, deep chemical peels or dermabrasion;
- To use aspirin or non-steroidal anti-inflammatory drugs (NSAIDs) within 2 weeks before the treatment or to take concomitant anticoagulant therapy, anti-platelet therapy, or to have a history of bleeding disorders;
- To use photosensitizing drugs (antidepressants, retinoids) within 2 weeks prior to the treatment.

Removal of Patients from Therapy or Assessment Investigational Plan. A subject was to be withdrawn from the study for any of the following reasons:

- Lost to follow-up;
- Withdrawal of consent;
- The Principal Investigator believed that for safety reasons (e.g., AE, concurrent illness) it was in the best interest of the subject to be withdrawn from study participation;
- The subject’s attending physician requested that the subject be withdrawn from the study;
- Lack of compliance to study procedures or poor visit attendance;
- A significant protocol deviation or violation.

### 2.3. Treatment

The decision to treat and the choice of needle/cannula, access point, technique, and volume of injection were made at the discretion of the treating physician. Treatment involved one deep administration of Neauvia Organic Stimulate during one treatment session. The deep anatomical plane selected was subcutaneous in accordance with the clinical correction and anatomic condition of the tissue layers. The product was delivered in the deep subcutaneous layer under an aseptic condition with a 27-gauge needle (Bolo Technique), in a single bolus where the clinical condition needed a vertical vector. In the case of a homogeneous and linear infiltration in the more superficial subcutaneous layer, the 22-gauge cannula (retrograde sliding injection technique) was preferred. The maximum quantity for the treated area was 1 mL.

### 2.4. Assessment

All patients were evaluated immediately after 1 month, 3 months, and 6 months after injection by the physician using moderate to severe age-related mid-face volume deficit according to the mid-face volume deficit scale (MFVDS) [27], a scale-specific validated 6-point assessment to quantify the results of the treatment, and, to complete the Global Aesthetic Improvement Scale (GAIS) [28], another 5-point scale that evaluates global aesthetic improvement in appearance is utilized.

We defined “Responder” as a patient who had at least a 1-point improvement in MFVDS from baseline. 

Moreover, for the clinical assessments, patients were asked to fill a satisfaction score module for the tested product, to evaluate their change in appearance after the treatment. This module was submitted to patients immediately after the treatment, 1 month after the treatment, 3 months after the treatment, and 6 months after the treatment. The satisfaction score was based on the visual analogue scale (VAS) [29,30]; the score is determined by measuring the distance on the 10 cm line between the “no satisfaction” and “high satisfaction” and the patient’s mark, providing a range of scores from 0–10. A higher score indicates greater satisfaction. The same module was filled by the PI or the CIT in order to evaluate the Investigator’s satisfaction with the treatment.

### 2.5. Data Collection

Background information, including standardized facial photographs, was collected prior to treatment. Details of the treatment session were recorded; these details included needle/cannula size, technique, and volume of product used. Safety outcomes and treatment adverse events retrieved from patient records were reported. All treated subjects were asked for details of any post-treatment adverse events they might have experienced.

To quantify the results obtained from the treatment, the mid-face volume deficit scale (MFVDS) [27] was used, a validated 6-point evaluation scale, which incorporates 5 categories, as follows: 0 = None; 1 = Minimum; 2 = Mild; 3 = Moderate; 4 = Significant and 5 = Severe.

A Global Aesthetic Improvement Scale (GAIS) [28] incorporating 5 categories was used to rate appearance, as follows: 1 = Very much improved; 2 = Much improved; 3 = Improved; 4 = No change; and 5 = Worse.

The satisfaction score was based on the visual analogue scale (VAS) [29,30]. It was determined by measuring the distance on the 10 cm line between “No satisfaction” and “High satisfaction”. Therefore, this score was calculated precisely by counting the values of the same, always from 0 to 10, and a higher result logically indicated greater satisfaction.

### 2.6. Statistical Analyses

All statistical analyses were performed using Jamovi software version 2.2.5 [31,32]. The software used to perform the statistical analysis, as well as the data management activities, is fully validated. The analysis was mainly descriptive, with quantitative variables expressed as means and standard deviations, and qualitative variables expressed as frequencies and percentages. Quantitative variables are compared by t-test for paired data. Frequencies and percentages are analyzed by the Chi-square test, while the comparison of non-parametric scales measured before and after the stimulate injection was performed by ANOVA for repeated measurement (Friedman Chi-square) and also by the Wilcoxon test.

## 3. Results

### 3.1. Disposition of Patients

Initially, 70 patients were enrolled, but 10 (14.29%) did not complete the study due to non-observance of the investigation rules, so they were excluded from the protocol.

### 3.2. Dosing

Patients received treatment with NS-26-1 injectable sub-dermal filler in one session on area zygomatic for correction. The total mean dose injected for all patients, in the zygomatic area, was 1 mL.

### 3.3. DemoGraph and Baseline Characteristics

A total of 60 patients were evaluated prospectively, comprising 56 females (93.33%) and 4 males (6.67%), with a mean age of 56.7 years (range: 26–70 years), as described in Table 1.

Prior to NS-26-1 injection, most patients (81.7%) were noted to have stage 3 of the defect to be corrected according to the severity of defects scale: (0 = None; 1 = Minimal; 2 = Mild; 3 = Moderate; 4 = Significant; and 5 = Severe), as reported in Table 2.

### 3.4. Effectiveness

We can state that 100% of the patients had an immediate response to the treatment with NS-26-1 injection, with a decrease over time also linked to the number of subjects who carried out the complete follow-up (Table 3). 

**Figure 2:**
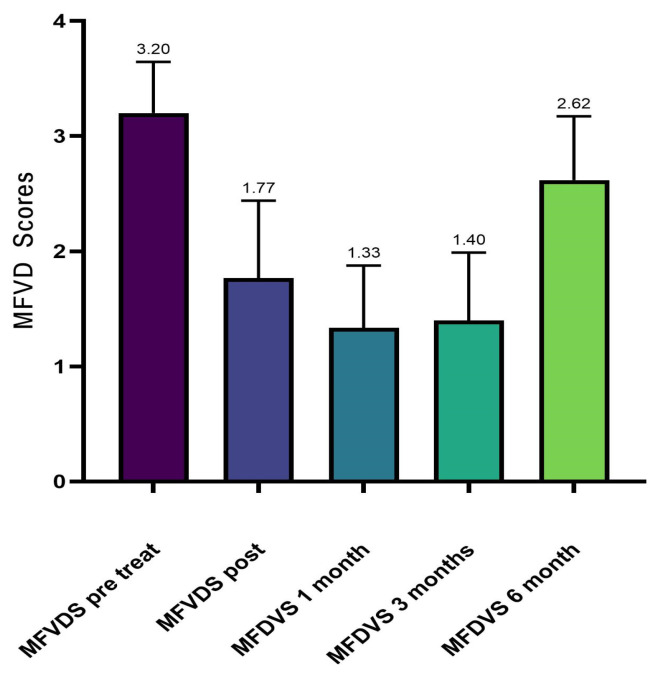
Average values of MFVDS improvement.

The results from the investigator assessment of the mid-face volume deficit scale (MFVDS) before, immediately after injection, and after 3 and 6 months are reported in Figure 2 and detailed in Table 4. 

**Figure 3:**
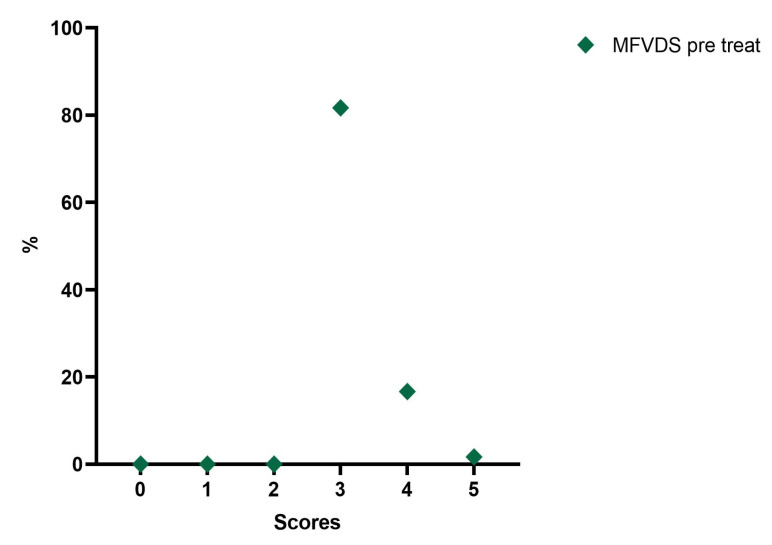
Distribution of MFVDS rate pre injection.

Analyzing the frequency of the MFVDS in the different time point evaluations the patients in the pretreatment condition showed this distribution (Table 5, Figure 3): “Moderate” 81.7%; “Significant” 16.7%; and “Severe” 1.7%.

**Figure 4:**
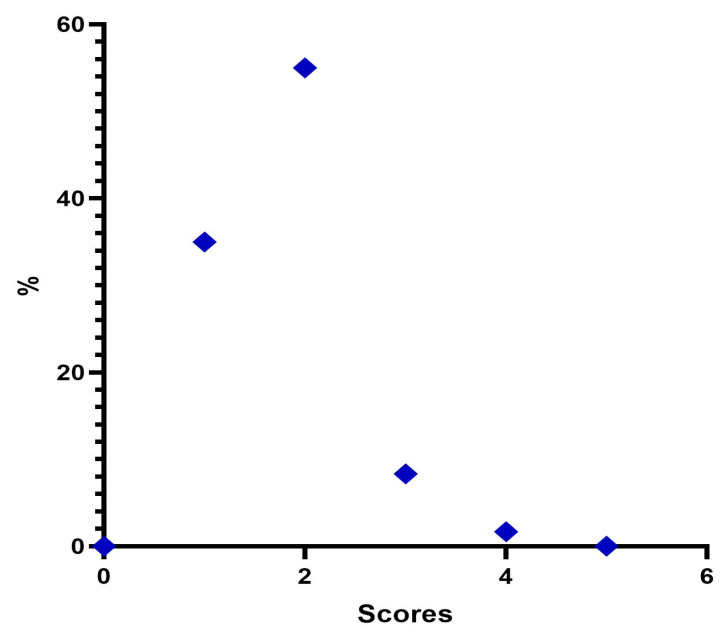
Distribution of MFVDS rate post injection.

Immediately after treatment, there is a change in the frequency distribution of the MFVDS where there was a “Minimal” 35%; “Mild” 90%; “Moderate” 8.3%; “Significant” 1.77%; and “Severe” 0% (Table 6, Figure 4). 

**Figure 5:**
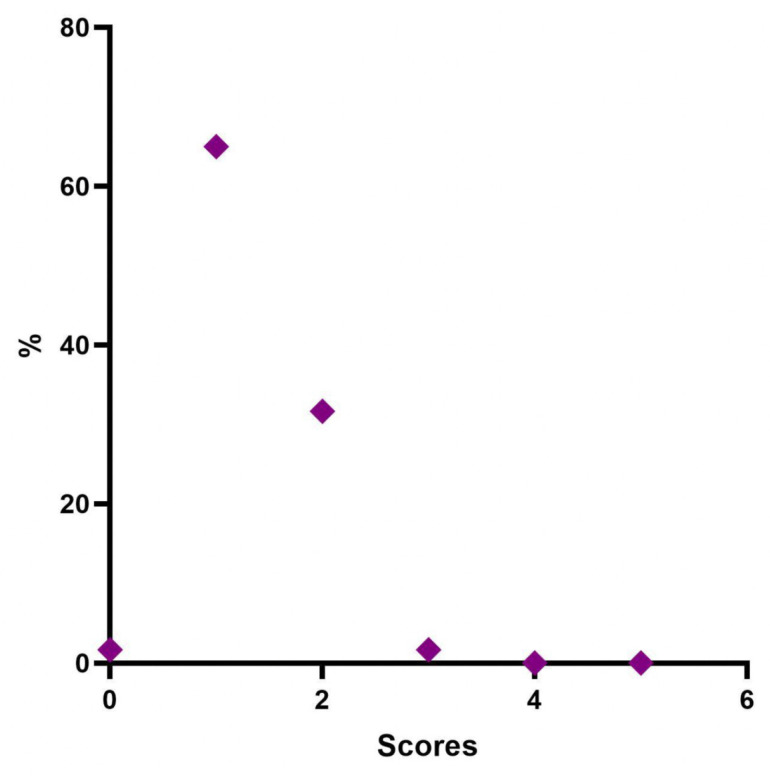
Distribution of MFVDS rate 1 month after injection.

Four weeks post-treatment 39 patients (65.0%) were considered “Minimal”, 19 patients (31.67%) were rated “Mild”, and 2 patients (3.3%) were considered “Moderate” (Table 7, Figure 5). 

**Figure 6:**
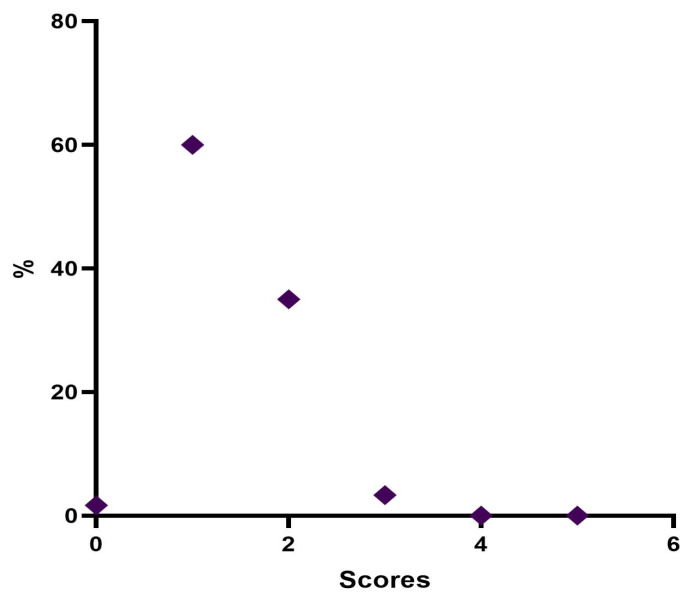
Distribution of MFVDS rate 3 months after injection.

Twelve weeks post treatment, 1 patient (1.7%) was considered “None”, 36 patients (60%) were rated “Minimal”, 21 patients (35%) were considered “Mild”, and 2 patients (3.3%) were rated “Moderate” (Table 8, Figure 6). 

**Figure 7:**
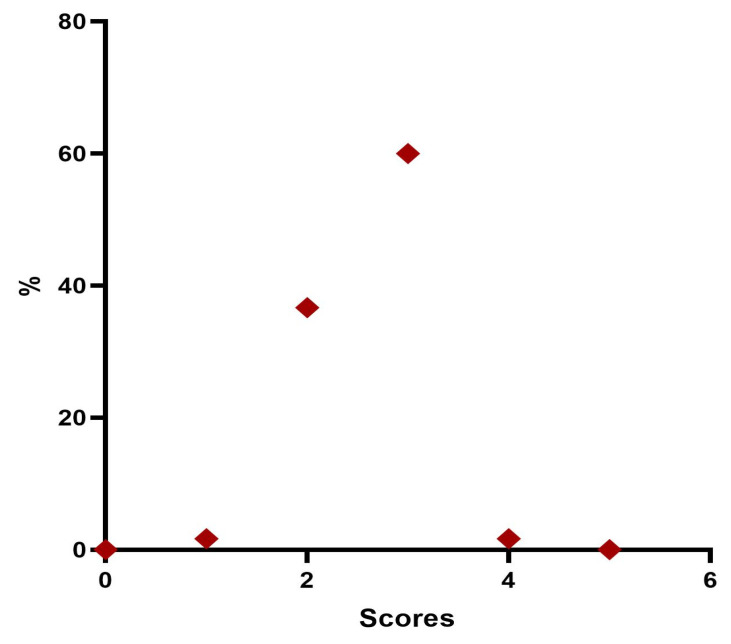
Distribution of MFVDS rate 6 months after injection.

Twenty-four weeks post treatment, 1 patient (1.7%) was considered “Minimal”, 22 patients (36.7%) were rated “Mild”, 36 patients (60%) were considered “Moderate”, and 1 patient (1.7%) were rated “Significant” (Table 9, Figure 7). 

**Figure 8:**
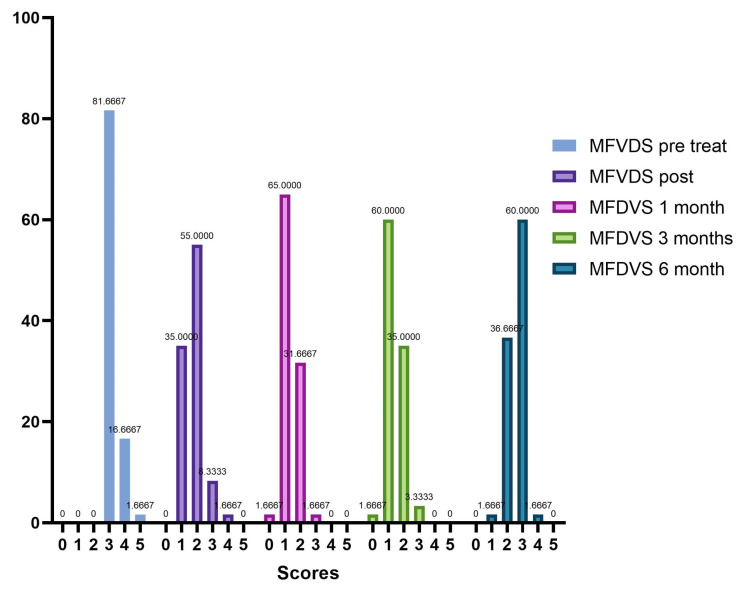
Trend of the frequencies of MFVDS on the different evaluation steps.

The trend in the frequency of MFVDS in the different time points is listed below in Figure 8.

The changes in the average values detected in the checks carried out immediately after the injection of the filler and in the subsequent checks are always statistically significant (ANOVA *p* < 0.001; Wilcoxon test *p* < 0.05) as reported in Table 10.

**Figure 9:**
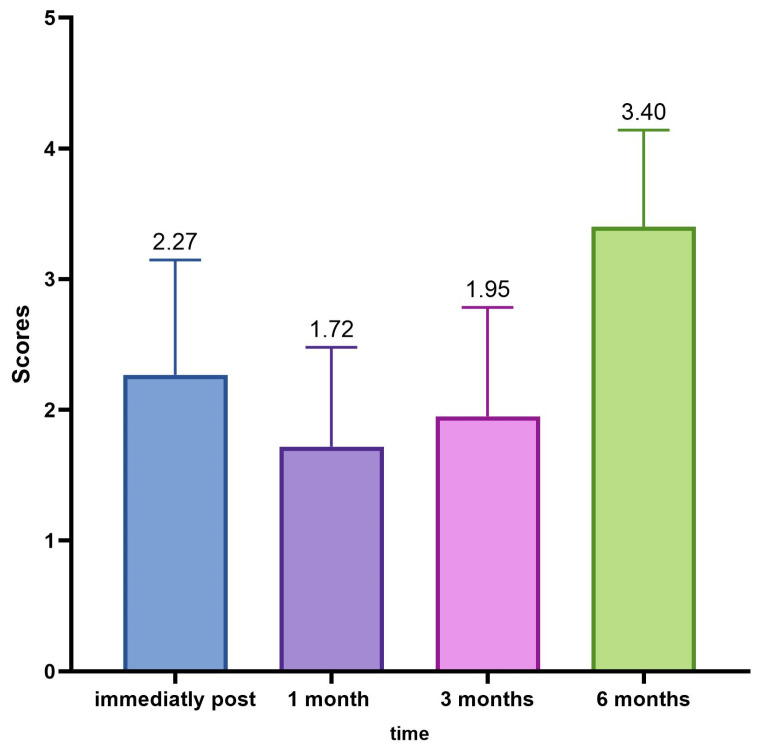
Average GAIS evaluation trend.

The average GAIS calculation of the population in the different evaluation time steps (0, 30, 90, 180 days) showed us an interesting trend with the maximum improvement of the correction in the time point of 30 and 90 days (Table 11, Figure 9). 

The detected changes in the mean values of the GAIS scores measured at each control are always statistically significant (ANOVA *p* < 0.001; Wilcoxon *p* < 0.05), as shown in Table 12.

Going deeply, results from the investigator assessment of GAIS at 4 weeks post treatment showed that most patients were “Very much improved” (28 patients—46%) or “Much improved” (21 patients—35%); 11 patients (18.3%) were rated as “Improved”. No patient was considered “Worse” following NS-26-1 treatment. 

Investigator GAIS assessment at 12 weeks post treatment highlighted that 21 patients (35%) were still considered “Very much improved”, 22 patients (36.7%) were rated as “Much improved”, and 16 patients (26.7%) were considered “Improved”. One patient (1.7%) was judged to be “No change” and no patients were considered “Worse” at this time point.

Investigator GAIS assessment at 24 weeks post treatment highlighted that 0 patients (0%) were still considered “Very much improved”, 9 patients (15%) were rated “Much improved”, and 18 patients (30%) were considered to be “Improved”. Thirty-three patients (55%) were judged to be “No change” and no patients were considered ‘Worse’ at this time point. 

**Figure 10:**
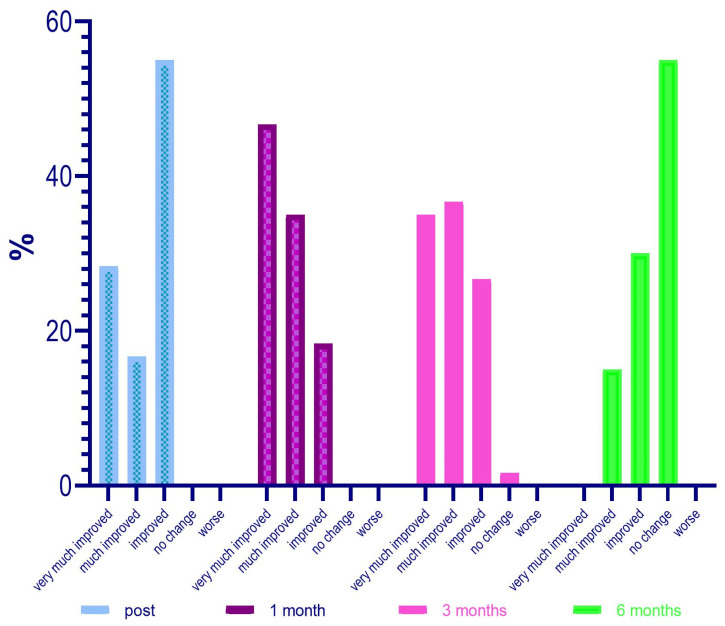
GAIS evaluation trend in the different time points.

The GAIS evaluation trend in the different time points is described in Figure 10. 

The satisfaction score was based on the visual analogue scale (VAS). The score was determined by measuring the distance on the 10 cm line between “No satisfaction” and “High satisfaction”; it followed that the patient’s or doctor’s score fell within a range of scores from 0 to 10. A higher score indicated greater satisfaction from one and a better evaluation from the other.

**Figure 11:**
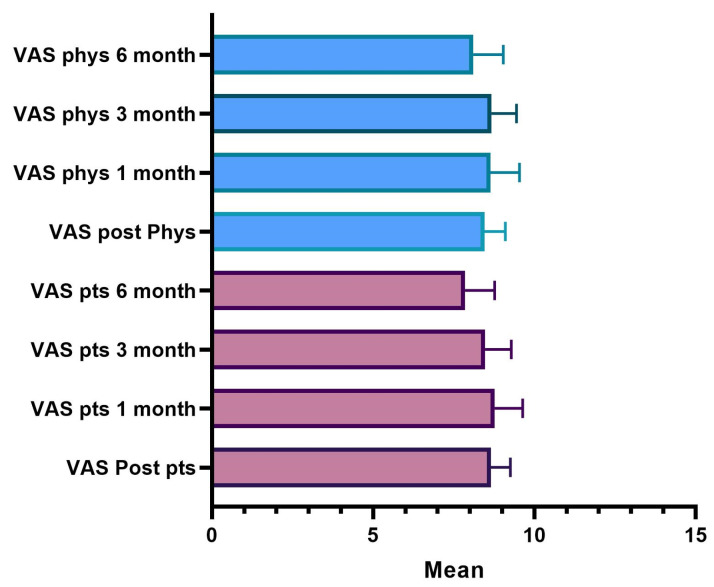
VAS satisfaction evaluation.

The average value of patient and doctor satisfaction in the various time points is shown in Figure 11; Table 13 shows the descriptive analysis of the VAS rate at the different points. 

## 4. Discussion

From the analysis of our data, the patients enrolled in the first instance were 70, but in the continuation of the study, 10 (14.29%) did not complete the whole procedure due to failure to comply with the rules of the survey. Therefore, ultimately, 60 subjects were evaluated prospectively, 56 of whom were female (93.33%) and 4 male (6.67%), with a mean age of 56.7 years (range: 26–70 years). In the initial and pre-injection phase of Neauvia Organic Stimulate, 49 patients (81.7%) presented a stage 3 (Moderate) defect to be corrected according to the defect severity scale. Ten subjects (16.7%) presented a stage 4 (Significant), while only one patient (1.7%) showed a stage 5 (Severe).

Our evaluation shows that 100% of patients had an immediate response to treatment with Neauvia Organic Stimulate injection, with a decrease over time also linked to the actual number of subjects who completed the follow-ups. All 60 patients responded to treatment as per protocol, and the response rate remained at 50% at one-month post-treatment and 35% at 3 months. Immediately after treatment, the frequency distribution of the MFVDS resulted as follows: 21 patients (35.0%) were classified as stage 1 “Minimal”, 33 (55.0%) were considered stage 2 “Mild”, 5 (8.3%) were definable as stage 3 “Moderate”, and only 1 (1.7%) was identifiable in stage 4 “Significant”. None belonged to stage 5 “Severe”.

After four weeks of treatment, the results suggested that 39 patients (65.0%) were classified as stage 1 “Minimal”, 19 (31.6%) were considered stage 2 “Mild”, and 2 (3.4%) were described as stage 3 “Moderate”. None belonged to stage 4 “Significant” or stage 5 “Severe”.

At the end of 12 weeks post treatment, it was possible to define that 1 patient (1.7%) was rated stage 0 “None”, 36 patients (60.0%) were classified stage 1 “Minimal”, 21 (34.9%) were considered stage 2 “Mild”, and 2 (3.4%) were definable as stage 3 “Moderate”. None belonged to stage 4 “Significant” or stage 5 “Severe”.

After 6 months from the injection treatment, the data defined that 1 patient (1.7%) was classified as stage 1 “Minimal”, 22 (36.6%) were considered stage 2 “Mild”, 36 (60.0%) were definable as stage 3 “Moderate”, and 1 (1.7%) was identifiable in stage 4 “Significant”. None belonged to stage 5 “Severe”.

And all the changes in the average values detected in the checks carried out immediately after the injection of the filler and in the subsequent checks are always statistically significant (ANOVA *p* < 0.001; Wilcoxon test *p* < 0.05).

Regarding the evaluation of the GAIS by the investigator, it was defined that at 4 weeks after treatment, 28 patients (46%) were “Very much improved”, 21 (35%) were “Much improved”, and 11 (18.3%) judged “Improved”. No subject was rated as “Worse” after treatment with Neauvia Organic Stimulate.

The investigator’s GAIS assessment at 12 weeks post treatment certified that 21 patients (35%) were still rated “Very much improved”, 22 (36.7%) were rated “Much improved”, and 16 (26.7%) rated “Improved”. One subject (1.7%) was classified as “No change” and none as “Worsened” at that time.

The investigator’s GAIS assessment at 24 weeks post treatment showed that no patients (0%) were yet considered “Very much improved”, while 9 (15%) could be defined as “Much improved”, and 18 (30%) as “Improved”. Thirty-three subjects (55%) had the status of “No change”; however, none were defined as “Worsened” at that precise moment.

The average GAIS calculation of the population in the various evaluation time phases (0, 30, 90, and 180 days) has highlighted an interesting trend with the maximum improvement of the correction in the time point of 30 and 90 days, and, in this case, the variations of the mean values of the scores of the GAIS scale, measured at each control, were always statistically significant (ANOVA *p* < 0.001; Wilcoxon *p* < 0.05).

The satisfaction score was based on the visual analogue scale (VAS). As already explained, the patient’s or investigator’s score fell within a range of scores from 0 to 10, from “No satisfaction” to “High satisfaction”. A higher score, of course, indicated greater satisfaction with one and a better evaluation of the other.

The results showed that 98.3% of the patients, immediately after the treatment, expressed a degree of satisfaction higher than 7; after one month, 85% still rated a score higher than 8, and after three months, 91.7% of the patients are still very satisfied. After six months, 56% of the subjects still expressed a VAS value greater than 8. Over the six months, the score of the evaluations recorded a lowering of the scores and the average value decreased statistically significantly.

A fully overlapping analysis can be done for physicians’ VAS assessments. The results define that 100% of the treatments, according to the doctor’s judgment immediately after the injection, showed a degree of satisfaction greater than 7; after one month 90% of the treatments were still satisfactory according to the doctor who assigns a VAS score greater than 8; three months after treatment 93% were still satisfied with a score greater than 8; and six months satisfaction with a score greater than 8 was present in 71% of cases. It is only after six months that there is a decrease in the score, the average value of which is statistically significant.

Although fillers are generally considered safe, some side effects such as bruising, redness, swelling, pain, tenderness, and itching may occur, and a low incidence of complications is reported in the literature [33]. The classification of filler complications can be made according to severity (mild, moderate, or severe), nature (ischemic and non-ischemic complications) or time of onset (early or late) [3,34].

Rohrich et al. [35] suggested classifying complications into early, late, and delayed, roughly defined as less than 14 days, 14 days to 1 year, and more than 1 year, respectively, as these timeframes are well related to the potential underlying etiology.

The facial vascular complication is one of the most serious and critical early complications in the use of fillers, induced by an interruption of the vascular supply to the area due to direct injury of the vessels, compression, and/or obstruction of the vessels (embolization) by the filler material [33].

No major side effects were recorded during the clinical trial.

## 5. Conclusions

In this post-market, observational, prospective, real-life, open-label, single-center, study, the Pegylated hyaluronic acid filler enriched with calcium hydroxyapatite prospective was shown to be effective for the correction of the disorder of the skin and subcutaneous tissue, and in particular in the mid-face volume deficit correction. The collected data demonstrate an effective mechanical effect of the pegylated polymeric acid matrix enriched with low concertation of calcium hydroxyapatite and in accordance with other evidence in vitro and in vivo, the mechanical support of the interstitial connective space improves the homestays of the anatomical layer rebalancing the physiological activity of the dermis cells. The possibility of the pegylated hydrogel matrix substances of different compositions opens a reflection for future use of these medical devices like a future scaffold for a timing drug delivering in local therapy of the same dermatologic pathology [36].

## Figures and Tables

**Table 1:** Description of the sample.

N	60
Missing	0
	AGE
Mean	56.7
Median	59.0
Standard deviation	10.0
Minimum	26
Maximum	70

**Table 2:** Frequencies of MFVDS pre injection.

Levels	Counts	% of Total	Cumulative %
3	49	81.7%	81.7%
4	10	16.7%	98.3%
5	1	1.7%	100.0%

**Table 3:** Percentage of responding patients as defined in the protocol.

MFVDS	n°	%
Responders pre vs. post	60	100
Responders post vs. 1 month	30	50
Responders post vs. 3 months	21	35
Responders post vs. 6 months	03	05

**Table 4:** Mean values of MFVDS scale.

	MFVDS Pre	MFVDS Post	MFVDS 1 Month	MFVDS 3 Months	MFVDS 6 Months
N	60	60	60	60	60
Missing	0	0	0	0	0
Mean	3.20	1.77	1.33	1.40	2.62
Median	3.00	2.00	1.00	1.00	3.00
Standard deviation	0.443	0.673	0.542	0.588	0.555
Minimum	3	1	0	0	1
Maximum	5	4	3	3	4

**Table 5:** Frequencies of MFVDS pre-injection.

Levels	Counts	% of Total	Cumulative %
3	49	81.7%	81.7%
4	10	16.7%	98.3%
5	1	1.7%	100.0%

**Table 6:** Frequencies of MFVDS immediately post injection.

Levels	Counts	% of Total	Cumulative %
1	21	35.0%	35.0%
2	33	55.0%	90.0%
3	5	8.3%	98.3%
4	1	1.7%	100.0%

**Table 7:** Frequencies of MFVDS 1 month after injection.

Levels	Counts	% of Total	Cumulative %
0	1	1.7%	1.7%
1	39	65.0%	66.7%
2	19	31.7%	98.3%
3	1	1.7%	100.0%

**Table 8:** Frequencies of MFVDS 3 months after injection.

Levels	Counts	% of Total	Cumulative %
0	1	1.7%	1.7%
1	36	60.0%	61.7%
2	21	35.0%	96.7%
3	2	3.3%	100.0%

**Table 9:** Frequencies of MFVDS 6 months after injection.

Levels	Counts	% of Total	Cumulative %
1	1	1.7%	1.7%
2	22	36.7%	38.3%
3	36	60.0%	98.3%
4	1	1.7%	100.0%

**Table 10:** Statistical significance of MFVDS rates measured at the different evaluation steps.

MFVDS	Post	1 Month	3 Months	6 Months	Overall ANOVA
Pre Wilcoxon	*p* < 0.05	*p* < 0.05	*p* < 0.05	*p* < 0.05	
Pre ANOVA–Chi Friedman	*p* < 0.001	*p* < 0.001	*p* < 0.001	*p* < 0.001	*p* < 0.001

**Table 11:** Mean values of GAIS Rate at the different evaluation steps.

	Immediately Post	1 Month	3 Months	6 Months
N	60	60	60	60
Missing	0	0	0	0
Mean	2.27	1.72	1.95	3.40
Median	3.00	2.00	2.00	4.00
Standard deviation	0.880	0.761	0.832	0.741
Minimum	1	1	1	2
Maximum	3	3	4	4

**Table 12:** Statistical significance of GAIS rates measured at the different evaluation steps.

GAIS	1 m	3 m	6 m	Overall ANOVA
Post Wilcoxon	*p* < 0.05	*p* < 0.05	*p* < 0.05	
1 m		*p* < 0.05	*p* < 0.05	
Post ANOVA–Chi Friedman	*p* < 0.001	*p* < 0.001	*p* < 0.001	*p* < 0.0010001
1 m		*p* < 0.001	*p* < 0.001	

**Table 13:** Description of VAS score for patients.

	Post Treatment	4 Weeks	12 Weeks	24 Weeks
N	60	60	60	60
Missing	0	0	0	0
Mean	8.65	8.77	8.47	7.85
Median	9.00	9.00	8.00	8.00
Standard deviation	0.606	0.871	0.812	0.917
Minimum	7	7	7	7
Maximum	10	10	10	10

## Data Availability

The data presented in this study are available on request from the corresponding author. The data are not publicly available due to GDPR compliance.
